# Towards reliable prediction of intraoperative hypotension: a cross-center evaluation of deep learning-based and MAP-derived methods

**DOI:** 10.1007/s10877-025-01357-0

**Published:** 2025-09-12

**Authors:** Nada Chaari, Greg Winski, Magnus Hallbäck, Niclas Lundström, Håkan Björne, Martin Jacobsson

**Affiliations:** 1 Department of Biomedical Engineering and Health Systems, KTH Royal Institute of Technology, Hälsovägen 11, Huddinge, 141 52 Sweden; 2 Department of Perioperative Medicine and Intensive Care, Karolinska University Hospital, Huddinge, 141 86 Sweden; 3 Department of Physiology and Pharmacology, Karolinska Institutet, Solnavägen 9, Solna, 171 77 Sweden; 4 Maquet Critical Care AB, Röntgenvägen 2, Solna, 171 06 Sweden

**Keywords:** Intraoperative hypotension, Arterial blood pressure, Deep convolutional neural network, Hypotension events, Model generalizability, MAP-derived methods

## Abstract

Intraoperative hypotension (IOH) is associated with an increased risk of heart and kidney complications. Although AI tools aim to predict IOH, their real-world reliability is often overstated due to biased data selection. This study introduces a framework to enhance reliability by: (1) including borderline blood pressure cases (65–75 mmHg, the “Gray Zone”), (2) comparing AI model to simple blood pressure threshold, and (3) validating across diverse surgical cohorts, centers and demographics. Using datasets from Karolinska University Hospital (Sweden) and VitalDB (Korea), we found AI model performs better than MAP threshold method in more ambiguous cases. In contrast, when hypotensive and non-hypotensive cases had clearly separated MAP values, both methods performed similarly well. Cross-validation revealed asymmetric generalizability: models trained on datasets containing more borderline (Gray Zone) cases generalized better to datasets with clearer class separation, whereas the reverse struggled. To ensure fair model comparison and reduce dataset-specific bias, we standardized the MAP difference between positive (hypotension) and negative (non-hypotension) samples at the time of prediction. This virtually eliminated the class separation and demonstrated that inflated performance in some datasets can be attributed to selection bias rather than true model generalizability. Age also influenced generalization: Cross-age validation revealed models trained on older patients generalized better to younger cohorts, whereas differences in ASA classification had minimal effect. These findings highlight the need for realistic validation to bridge the gap between AI research and clinical practice.

**Supplementary Information:**

The online version contains supplementary material available at 10.1007/s10877-025-01357-0.

## Introduction

Intraoperative hypotension (IOH), defined as a significant decrease in blood pressure during surgery, has been associated with postoperative complications such as acute kidney injury (AKI), myocardial injury, and increased mortality [25, 30]. This association may relate to impaired organ perfusion and an oxygen supply-demand mismatch during hypotensive episodes, which could contribute to tissue injury [3, 31]. Current evidence suggests that reducing the depth and duration of IOH may correlate with lower postoperative risks. Current monitoring systems enable hemodynamic tracking to manage IOH and guide treatment. Although clinicians may anticipate many IOH episodes due to their temporal relationship with surgical interventions or hemodynamic developments, rapid blood pressure changes may still challenge purely reactive management. Emerging predictive monitoring methods, which identify physiological patterns preceding instability, could offer clinicians additional time to consider preventive measures, potentially supporting efforts to reduce postoperative organ dysfunction.

Artificial intelligence (AI) models have shown promise in anticipating IOH in real-time, allowing clinicians to intervene proactively and maintain hemodynamic stability [22]. Additionally, these AI models have been helpful in forecasting and mitigating complications related to hypotension, such as myocardial infarction and AKI [20, 34]. One notable AI model is the Hypotension Prediction Index (HPI) [11], which uses a logistic regression algorithm based on arterial blood pressure (ABP) waveform features to predict hypotension in real-time. In this context, several studies have evaluated the predictive ability of the HPI by validating its performance across diverse surgical populations [22, 26].

Although these studies indicate that the HPI can effectively anticipate hypotensive episodes, its benefits must be carefully weighed against well-established hemodynamic parameters, as its comparative effectiveness remains under active debate. Mulder et al. [23] found no significant difference between HPI and Mean Arterial Pressure (MAP)-based predictions for predicting IOH during non-cardiac surgery, consistent with earlier MAP-derived methods [14]. Massari et al. [21] and Yang et al. [33] reported identical predictive performance between HPI and MAP thresholds across multiple clinical scenarios. Multiple studies demonstrate that HPI strongly correlates with concurrent MAP values [7, 9, 15]. They demonstrated comparable performance when evaluated across the whole blood pressure range.

The validity of HPI validation studies, including the original model development [11], has been questioned due to methodological selection bias. These studies artificially excluded MAP values within the clinically contested 65–75 mmHg range–commonly referred to as the “Gray Zone”–by defining non-hypotension events strictly as MAP $>75$ mmHg. This exclusion enforced binary class separation (hypotensive/non-hypotensive) but created an artificial hemodynamic vacuum, ignoring the transitional phase where diagnostic uncertainty is the highest [8, 28]. By omitting this intermediate range, prior work likely inflated predictive performance by avoiding diagnostically ambiguous cases, thereby introducing a systemic bias favoring idealized scenarios over clinically realistic conditions.

In addition to HPI, other AI models, particularly those based on deep learning (DL), have also demonstrated significant capabilities in predicting IOH. For instance, Lee et al. [19] applied a convolutional neural network (CNN)-based architecture to analyze multichannel waveforms from ABP, electrocardiogram (ECG), photoplethysmography, and capnography data, showcasing how incorporating multiple data sources can enhance prediction ability, although not a lot. Similarly, Jo et al. [16] proposed a model based on deep residual network (ResNet) [12], a more advanced CNN architecture, which used ABP, ECG, and electroencephalogram (EEG) data to improve predictions of hypotensive events. Furthermore, a comparative study of recurrent neural network (RNN) and CNN-based models reported that a fusion of both architectures slightly outperforms the use of either one alone [4].

Despite advances in AI-driven hypotension prediction, existing models exhibit notable limitations that necessitate further investigation. First, numerous DL models are developed and validated on single-source datasets, predominantly derived from a specific clinical environment, failing to account for variability in patient demographics, surgical practices, and hospital-specific protocols. This lack of external validation limits generalizability and raises concerns about reliability in real-world settings. Second, persistent methodological biases create an unrealistic separation between hypotensive and non-hypotensive states, leading to overoptimistic estimates of model utility in clinical practice. Together, these issues–narrow validation cohorts and biased data selection–compromise the reliability of existing prediction methods, limiting their translation into actionable clinical tools.

We propose a three-pronged framework to enhance hypotension prediction reliability: 1) Redefining the hypotension/non-hypotension events selection to include the 65–75 mmHg Gray Zone, reducing artificial class separation; 2) Conducting a Head-to-head comparison between CNN-based model and MAP threshold; 3) Systematically validating generalizability across centers (different hospitals), surgical cohorts (diverse surgical procedures), and demographics. We organize the remainder of this paper as follows: Section 2 details the used data sets, the data collection, preprocessing, and selection. Section 3 outlines the evaluation framework for comparing DL model and MAP method, investigates the generalizability of the CNN-based classifier, and examines the impact of demographic and physiological factors on its performance. Section 4 presents model performance results, while Sect. 5 discusses their implications. Finally, Sect. 6 concludes the study and suggests future directions for early IOH prediction research.

## Material

Approval for this retrospective study was obtained from the Swedish Ethical Review Authority (registration no.: 2021-03073, 2022-05955-02, 2022-06063-01, and 2023-07000-02). The board waived the need for explicit patient consent to the collection and use of the data. To ensure the rigor and reproducibility of our study, we adhered to the TRIPOD-AI guidelines [5].

### Data collection

We used two data sets sourced from different hospitals, both selected based on shared core inclusion criteria: general anesthesia, age over 18, and surgery duration of more than 2 hours to ensure a sufficient number of hypotension events, as these patient populations are more prone to hypotension.

**Karolinska dataset:** This dataset consists of 457 patients who underwent abdominal surgery at Karolinska University Hospital, Sweden, between Jun 2023 and Feb 2024. The surgeries were obtained at the two Karolinska University Hospital sites: Huddinge and Solna. The ages of the patients ranged from 19 to 85 years. Continuous recordings were obtained from the catheter placement until removal, covering the intraoperative and postoperative periods. Data were sampled at 125 Hz, using a radial catheter connected to a Philips Intellivue MX800 bedside monitor.

**VitalBD dataset:** The second data set is sourced from an open-source repository [18, 29] and encompasses intraoperative biosignals and clinical information from 6,388 surgical patients at Seoul National University Hospital, Korea from Aug 2016 to Jun 2017. VitalDB data was initially recorded at 500 Hz and subsequently downsampled to 125 Hz using an FFT-based method (SciPy resample) to align with the sampling frequency of the Karolinska dataset.

**Fig. 1:**
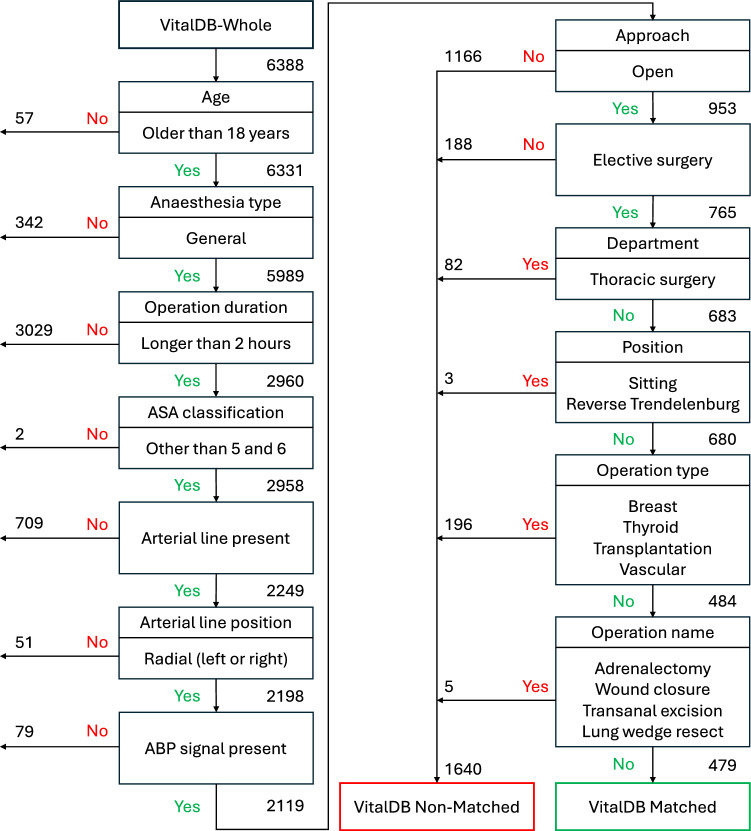
VitalDB dataset selection for cross-center and cross-cohort validations: This resulted in two cohorts: VDB Matched, comprising patients with similar surgical procedures to the Karolinska data set, and VDB Non-Matched, encompassing patients with other surgical procedures

To conduct cross-center and cross-cohort validation, we carefully assessed patient selection criteria and extracted two distinct cohorts from the VitalDB dataset. For the first cohort, aimed at cross-center validation, we selected patients based on additional criteria that mirror those of the Karolinska cohort. The details of these selection criteria are illustrated in Fig 1. This process resulted in a cohort of 479 patients called “VDB Matched”. However, this number was reduced to 438 patients after excluding those with less than 90% clean ABP data throughout the entire surgery duration, Table 1. For the second cohort, which was intended for different cohort validation, we used the remaining patients in the VitalDB data set who did not meet the criteria for the previous cross-center validation experiment. This subset, referred to as “VDB Non-Matched”, includes 1,640 patients, Fig 1, and embodies a broader spectrum of surgical characteristics, encompassing variations in surgical approaches, departments, positions, types, and names. After applying the 90% clean ABP data criterion, the final size of the VDB Non-Matched cohort was reduced to 1,526 patients, Table 1.

Table 1 summarizes key demographics and clinical characteristics for each dataset, including age, sex, body mass index (BMI), American Society of Anesthesiologists (ASA) classification, surgery department, approach, emergency operation status, total operation duration (in hours), mean MAP during surgery (mean MAP in mmHg), and the fraction of operation time with MAP below 65 mmHg (Fraction of MAP < 65 in $\%$). In addition, the analysis reports the number of hypotension events per patient and hypotension severity metrics. These include total hypotension duration (minutes/patient), representing the total time a patient experiences hypotension, and hypotension event duration (minutes/event), indicating the average duration of individual hypotension events. The area under threshold (AUT, mmHg$\cdot$minutes) measures the depth of MAP below 65 mmHg multiplied by the time spent below this threshold, while the time-weighted average (TWA, mmHg) normalizes AUT by the total operation duration. Another metric, $\Delta \text {Mean}$, measures the difference between the mean MAP values of positive and negative data point distributions, each representing a 20-second ABP waveform segment extracted within a dynamically framed Observation Window. Positive points are associated with hypotension events occurring in the subsequent Prediction Window, while negative points have no hypotensive events in the Observation, Prediction, or Slack Windows. $\Delta \text {Mean}$ metric is visualized in Fig. 3 (left panel) for Karolinska, VDB Matched, and VDB Non-Matched datasets.

**Table 1:** Patient characteristics for Karolinska, VDB Matched, and VDB Non-Matched. Values shown as mean ± standard deviation), or as count (fraction in %)

Dataset	Karolinska	VDB Matched	VDB Non-Matched
Number of patients	419	438	1526
Age (years):	63.52 ± 12.41	61.82 ± 12.16	58.83 ± 14.22
Sex			
Female	240 (57.3%)	168 (38.4%)	599 (39.3%)
Male	179 (42.7%)	270 (61.6%)	927 (60.7%)
Height (cm)	170.47 ± 9.32	162.64 ± 8.55	163.58 ± 8.68
Weight (kg)	76.07 ± 16.22	60.75 ± 11.77	62.01 ± 11.24
BMI	26.1 ± 4.9	22.9 ± 3.62	23.1 ± 3.38
ASA classification			
1	28 (6.7%)	60 (13.7%)	346 (22.7%)
2	218 (52.0%)	327 (74.7%)	894 (58.6%)
3	168 (40.1%)	44 (10.0%)	237 (15.5%)
4	5 (1.2%)	2 (0.5%)	9 (0.6%)
Unknown	0 (0.0%)	5 (1.1%)	40 (2.6%)
Hypertension	183 (43.7%)	163 (37.2%)	536 (35.1%)
Diabetes	66 (15.8%)	75 (17.1%)	183 (12.0%)
Pulmonary disease	88 (21.0%)	114 (26.0%)	250 (16.4%)
Hemoglobin (g/l)	125.78 ± 17.0	125.93 ± 19.75	128.95 ± 20.11
Glucose (mmol/l)	7.52 ± 3.57	6.94 ± 2.81	6.55 ± 2.35
Albumin (g/l)	34.65 ± 4.67	39.14 ± 4.87	40.87 ± 4.92
Creatinine (micromol/l)	78.78 ± 50.66	72.22 ± 20.89	124.09 ± 200.43
Sodium (mmol/l)	139.59 ± 2.82	140.03 ± 2.67	140.02 ± 2.95
Potassium (mmol/l)	3.99 ± 0.37	4.18 ± 0.36	4.21 ± 0.42
Department			
Thoracic	0 (0.0%)	0 (0.0%)	462 (30.3%)
Urology	25 (6.0%)	2 (0.5%)	48 (3.1%)
General surgery	297 (70.8%)	416 (95.0%)	969 (63.5%)
Gynecology	97 (23.2%)	20 (4.5%)	47 (3.1%)
Approach			
Open	419 (100.0%)	438 (100.0%)	412 (27.0%)
Videoscopic	0 (0.0%)	0 (0.0%)	965 (63.2%)
Robotic	0 (0.0%)	0 (0.0%)	149 (9.8%)
Elective surgery	419 (100.0%)	438 (100.0%)	1327 (87.0%)
Total surgery duration (h):	5.07 ± 2.5	4.05 ± 1.58	3.62 ± 1.45
Mean MAP (mmHg)	74.42 ± 4.33	82.08 ± 9.27	83.78 ± 9.58
Hypotension prevalence	390 (93.1%)	299 (68.3%)	789 (51.7%)
Number of hypotension events	7.69 ± 6.57	4.45 ± 6.33	3.28 ± 6.41
Total hypotension duration (min)	22.85 ± 23.78	15.91 ± 26.11	12.32 ± 28.73
Time fraction in hypotension (%)	8.09 ± 7.74	6.62 ± 9.97	5.42 ± 11.33
Hypotension event duration (min)	2.6 ± 1.3	2.21 ± 2.07	1.64 ± 2.08
AUT (mmHg$\cdot$min)	13.09 ± 8.19	13.44 ± 15.07	8.88 ± 13.14
TWA (mmHg)	0.05 ± 0.05	0.06 ± 0.08	0.04 ± 0.07
$\Delta \text {Mean}$ (mmHg)	4.98	16.33	18.94

BMI = Body mass index, ASA = American Society of Anesthesiologists, MAP = Mean arterial pressure, AUT = Area under threshold, TWA = Time-weighted average, $\Delta \text {Mean}$ = The difference between the mean MAP values of the positive and negative data point distributions

### Data preprocessing

The ABP waveforms were preprocessed to remove noise, identify systolic and diastolic peaks, clean beat-to-beat artifacts, and calculate the Mean Arterial Pressure (MAP). Data filtering ensured that at least 90 % of clean ABP signals were available throughout the surgery duration. After preprocessing, the final patient sample sizes were 419 for the Karolinska dataset, 438 for the VDB Matched dataset, and 1,526 for the VDB Non-Matched dataset. These preprocessing steps are essential for ensuring data quality and reliability. A detailed description of the preprocessing pipeline, including specific algorithms and parameter settings, is provided in the supplementary material (section S1).

### Data point selection

We introduce a revised data selection strategy for early hypotension prediction using preprocessed ABP signals. Prior works [2, 32] typically adopt fixed sliding windows with deterministic strides (e.g., 1–2 minutes) and define labels according to a predetermined lead window before the onset of hypotension. While methodically consistent, these approaches risk introducing temporal correlations between adjacent samples, rigid framing assumptions, and label ambiguity–particularly when hypotensive events occur near the edges of the prediction horizon.

**Fig. 2:**
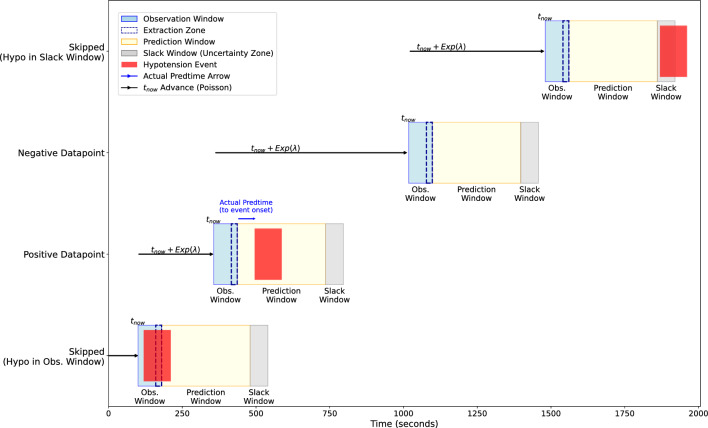
Dynamic Time-Sliding Window framing: This figure visualizes a Poisson-based sliding window for extracting physiological data points. Each segment advances dynamically from the previous one by $\Delta t$ (randomly sampled from an exponential distribution, capped at 3 minutes). At each $t_{now}$, the process involves: 1) Observation Window (1 min 20 secs): Data observed from $t_{now}$ and includes a Data Extraction Zone starting after a 1 min offset. 2) Prediction Window (5 min): Period for event prediction, following the Observation Window. 3) Slack Window (1 min): An ’uncertainty’ zone following the Prediction Window (totaling 5-6 min from prediction start), used to filter nearby events. Data point labeling logic: Positive: Labeled if a hypotensive event occurs in the Prediction Window. Negative: Labeled if no event occurs in Observation, Prediction, or Slack Windows. Skipped (Hypo in Obs. Window): If an event occurs within the Observation Window, the data point is removed from the dataset. Skipped (Hypo in Slack Window): If an event occurs within the Slack Window, a negative data point is skipped (to ignore negative points immediately preceding a close event)

To address these limitations, we adopt a stochastic, Poisson process-based sliding window framework. Candidate time points $t_{now}$ are sampled dynamically from an exponential distribution with rate $\lambda = 1/3$ min (mean inter-sample interval of 3 minutes), capped to prevent oversampling. Data points are extracted from the entire surgery time. Each sampled point $t_{now}$ anchors a structured framing scheme consisting of three consecutive windows: (1) a 1-minute 20-second Observation Window; (2) a 5-minute Prediction Window; and (3) a 1-minute Slack Window. This design ensures temporal independence between samples and improves training diversity, while also enforcing a forward-facing prediction model that aligns more realistically with clinical decision-making timelines, see Fig. 2.

The Observation Window begins at $t_{now}$ and serves two purposes: the first minute ensures a hypotension-free baseline, and the final 20 seconds are extracted as model input. The subsequent Prediction Window captures whether the onset of a hypotensive event–defined as MAP < 65 mmHg for at least one minute [11, 25]–occurs within the next 5 minutes. If so, the data point is labeled as positive. This inclusive labeling allows any event onset between $t_{now}$+1m20s and $t_{now}$+6m20s, rather than enforcing a fixed offset (e.g., exactly 5 minutes before onset), which limits clinical utility and can discard valid late warning cases [32].

Unlike some prior frameworks (e.g., [2, 32]) that enforce a fixed lead window (or lead time), our structure omits it. Although intended to ensure predictive relevance, a lead window can mislabel points shortly before a hypotension event as negative when the event actually occurs within the lead window. The lead window likely stems from the work by Lauritsen et al. [17], which is primarily about sepsis–an event that rarely repeats itself within a short time period in a way that hypotension can do.

To mitigate edge-labeling errors, we append a 1-minute Slack Window immediately after the Prediction Window. This ’uncertainty’ zone is not used for prediction but is critical for filtering near-onset events. For example, if a hypotensive event begins at $t_{now}$+6m21s–just beyond the 5-minute Prediction Window–labeling the point as negative would unfairly penalize early alarms. Instead, we exclude such points to avoid misleading the model. Without this, models may overlook high-risk patterns that emerge slightly earlier–potentially reducing clinical usefulness.

We also explicitly exclude any data point where a hypotensive event is already active anywhere during the Observation Window. This avoids training the model on signals already reflecting hypotension, which could bias the model toward detecting ongoing events rather than predicting future ones. Conversely, a data point is labeled as negative only if no hypotension occurs in both the Prediction or Slack Windows. This strict criterion ensures that negative samples truly represent periods of physiological stability, reducing false reassurance and allowing the model to better distinguish borderline pre-event dynamics, such as MAP values in the 65–75 mmHg range.

Our framing avoids the limitations of lead-window heuristics–commonly set between 2 and 15 minutes in prior work [2, 32], which may exclude informative pre-onset conditions. By eliminating arbitrary lead windows and introducing a Slack Window instead, we better balance inclusiveness and specificity. The extracted 20-second ABP segments are temporally decoupled from the event onset, maintaining clean labeling boundaries. This helps capture a wider range of subtle physiological trends preceding hypotension, supporting more faithful learning of early-warning signals.

**Fig. 3:**
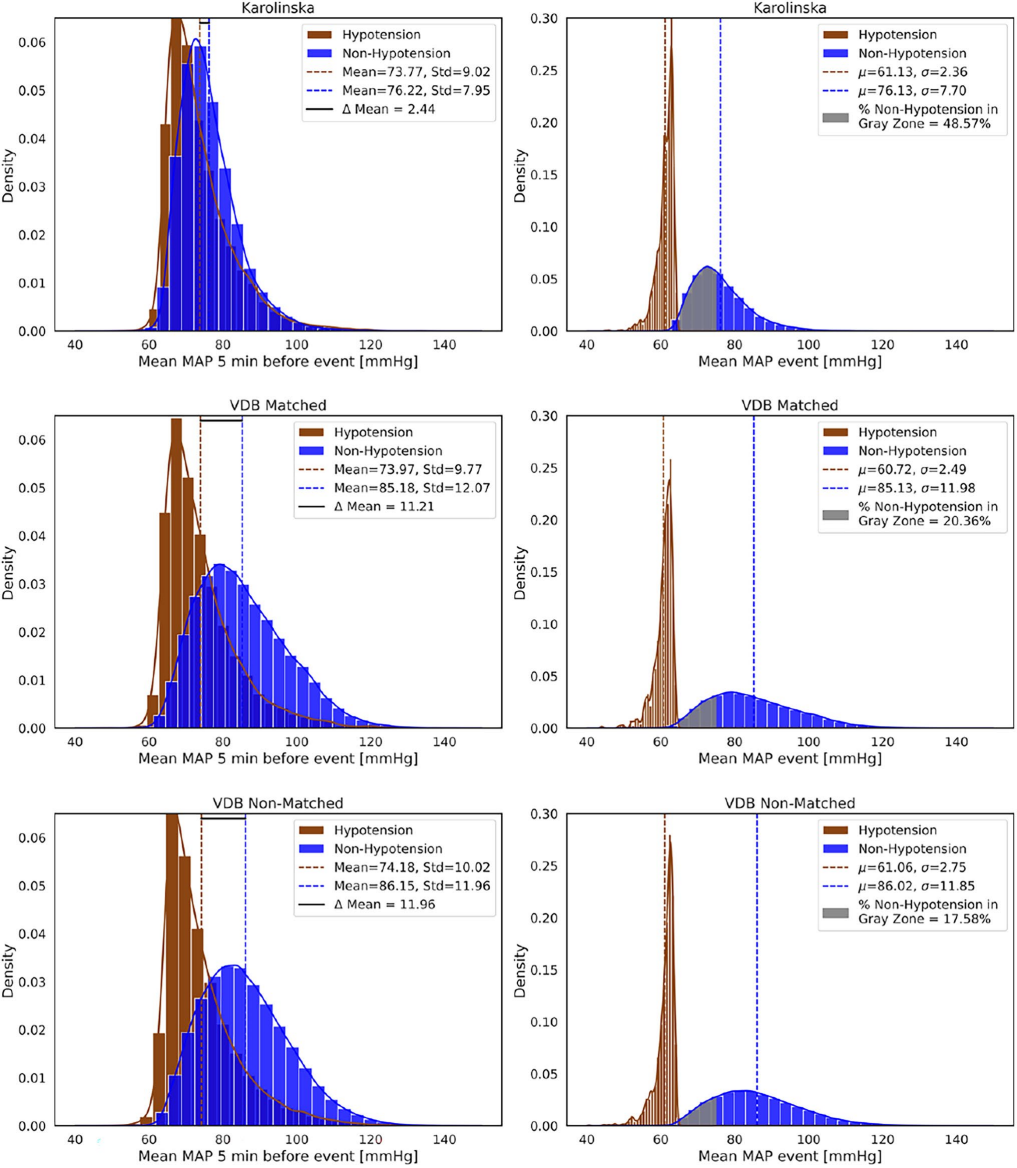
On the left panel of the figure is the distribution of the mean MAP values for positive and negative data points extracted at the prediction time of hypotension and non-hypotension events for the Karolinska, VDB Matched, and VDB Non-Matched datasets. On the right panel is the distribution of the mean MAP values during hypotension and non-hypotension events for the Karolinska, VDB Matched, and VDB Non-Matched datasets

To analyze the distribution of MAP values within these segments, we calculated the mean MAP for each 20-second window. Figure 3 (left panels) shows the distribution of mean MAP values for positive and negative data points across the Karolinska, VDB Matched, and VDB Non-Matched datasets. Notably, the mean MAP of negative data points–those predicting non-hypotension events–is lower in the Karolinska dataset (76.22 mmHg) compared to VDB Matched and Non-Matched datasets (ranging from 86.15 to 88.15 mmHg). In contrast, the mean MAP values of positive data points (hypotension events) are similar across all three datasets (approximately 74 mmHg). This results in a narrower separation between positive and negative classes of mean MAP values in the Karolinska dataset ($\Delta \text {Mean}$ = 2.44 mmHg) compared to VDB Matched and Non-Matched datasets ($\Delta \text {Mean}$ = 11.21–11.96 mmHg).

Figure 3 (right panels) illustrates the distribution of MAP values averaged over their respective 1-minute windows for hypotension and non-hypotension events across the Karolinska, VDB Matched, and VDB Non-Matched datasets. Notably, a higher proportion of non-hypotension events fall within the 65–75 mmHg range–denoted as the ”Gray Zone”–in the Karolinska dataset (48.57$\%$) compared to the VDB Matched and Non-Matched datasets (17.58–20.36$\%$). This indicates that our selection process for non-hypotension events did not explicitly exclude the Gray Zone; in fact, nearly half of the non-hypotension events in Karolinska fall within that range. This reduces the selection bias that has been present in previous studies (e.g., [11]). Another interpretation is that the number of non-hypotension events with MAP values between 65 and 75 mmHg is two to three times higher in the Karolinska dataset compared to VDB datasets. This suggests that overall MAP levels tend to be lower in Karolinska and that the Hemodynamic management strategy may aim to maintain MAP closer to 75 mmHg, rather than keeping it substantially above this threshold.

Although all data points at the time of prediction falling within a hypotension period were excluded for both hypotension and non-hypotension events, it is important to note that hypotensive periods lasting less than one minute are not classified as hypotension events under our selection strategy. As a result, brief drops in MAP may still occur within the 20-second segments surrounding both positive and negative prediction points. Consequently, the mean MAP values calculated over these segments can occasionally fall below 65 mmHg–even for data points labeled as non-hypotension. This behavior is illustrated in the supplementary material (Fig. S1), where a small number of both positive and negative data points show mean MAP values below 65 mmHg, as reflected by overlapping dots on the x-axis.

## Methodology

### Evaluation between CNN-based and MAP threshold methods

We conduct a comparative evaluation of DL models, specifically a CNN-based architecture, and MAP threshold method across the Karolinska, VDB Matched, and VDB Non-Matched datasets. We considered several DL models, including those proposed by Choe et al. [4], Jo et al. [16], and Jacobsson et al. [13]. These models, based on RNN-CNN, ResNet, and Inception-v4 architectures respectively, exhibited comparable performance to the CNN model by Lee et al. [19]. Given this similarity and to streamline our analysis, we proceeded with the Lee et al. model for our subsequent evaluations.

**CNN-based Model**: We employ the CNN-based model by Lee et al. [19], adapted for unidimensional signals. The supplementary material (Section S3) and the Implementation section contain details on the model architecture and training. The CNN processes 20-second ABP segments extracted at the prediction time of the event and predicts the probability of a hypotension or non-hypotension event. Predictions are compared to ground truth labels.

**MAP threshold (MAPthr)** [6]: This method evaluates predefined MAP thresholds (e.g., 55–85 mmHg) selected to cover the possible MAP values at which hypotension or non-hypotension events may occur 5 minutes later. The mean MAP of the 20-second segment, extracted at the prediction time of the event, is compared to these thresholds. If the mean MAP exceeds a threshold, the event is classified as non-hypotension or hypotension.

### Evaluation framework using CNN-based model

Additionally, a multi-faceted evaluation framework is implemented to assess the generalizability of the CNN model, addressing variability in clinical settings, surgical procedures, and patient demographics (e.g., age, ASA classification, and mean MAP). This approach provides a comprehensive assessment of model robustness and clinical applicability.

**Cross-center validation**: To evaluate the generalizability across different clinical settings, we performed cross-center validation using datasets (Karolinska vs. VDB Matched) from different hospitals with similar surgical procedures. The model is trained on one dataset and tested on the other.

**Cross-cohort validation**: To evaluate generalizability across datasets with different surgical procedures, we performed cross-center validation using datasets (e.g., Karolinska or VDB Matched vs. VDB Non-Matched). The model is trained on one cohort and tested on another.

**Cross-age**: To assess age-related variability, we combine the Karolinska dataset with both VDB Matched and VDB Non-Matched datasets, then divide into three age groups: the young cohort (18-55 years, 1079 patients) and the old cohort (67+ years, 783 patients). The middle-aged group (56-66 years) is excluded to maximize group separation while maintaining balance. Models are trained on one group and tested on the other.

**Mean MAP**: Significant differences in the mean MAP difference ($\Delta \text {Mean}$) between positive and negative data points were observed across datasets, which could influence model performance, as shown in Fig. 3 (left panel). We implemented a standardization process across datasets to address the potential impact of mean MAP variability on generalizability tests. This was achieved through a stratified sampling approach, where data points were binned based on their MAP values, and an equal number of samples were randomly selected from each bin for each class (positive and negative). This approach compares baseline conditions and controlled $\Delta \text {Mean}$ settings.

**ASA classification**: Differences in the distribution of ASA classification across datasets could impact model performance, as detailed in Table 1. To evaluate the impact of ASA classification, we conducted an experiment focusing exclusively on patients with ASA classification = 2, as this group represented the largest proportion of patients across all datasets. This analysis directly compares the baseline performance (including all ASA classifications) with the controlled setting, where only patients with ASA classification = 2 are considered.

### Evaluation metrics

To ensure reproducible results, we conduct a 5-fold cross-validation approach, splitting the data into training (70$\%$), validation (10$\%$), and testing (20$\%$) subsets while ensuring that data from the same patient was not split across different folds. Despite the augmentation strategy applied to the positive class, the dataset remained imbalanced, with a predominance of negative samples. To mitigate class imbalance during training, we applied class-balanced weighting in the CNN loss function, increasing the influence of positive samples. For evaluation, we reported a comprehensive set of metrics including AUC-ROC, AUC-PR, accuracy, sensitivity, specificity, positive predictive value (PPV), negative predictive value (NPV), and F1-score, all averaged across the 5 folds with 95% confidence intervals.

We acknowledge that AUC-PR is influenced by class prevalence, and while it provides insights into the trade-off between precision and recall, particularly valuable in settings with rare positive cases, it must be interpreted in light of class imbalance. Similarly, AUC-ROC, while commonly used, can sometimes overestimate performance in imbalanced contexts by emphasizing the true negative rate. Therefore, we report both AUC-ROC and AUC-PR, along with threshold-dependent metrics, such as PPV and F1-score, to provide a balanced and transparent view of model performance. Our evaluation strategy aims to account for both sensitivity and precision, especially under imbalanced data distributions.

### Implementation

VitalDB signals were first downsampled to 125 Hz using discrete Fourier transform-based resampling. ABP peaks were detected using Gaussian derivative filters and the Shannon energy envelogram [24]. Noise and artifacts (e.g., high-frequency noise, square waves) were removed beat-by-beat based on the signal abnormality index (see Table S1 in the supplementary). Data point selection followed a Poisson-based sliding window strategy, with a 1:20 min observation window, a 5-min prediction window, and a 1-min slack window. A data point was labeled positive if a hypotension event started within the prediction window. Negative points were randomly sampled with no hypotension during the prediction or slack periods.

The prediction model [19] is a 7-layer CNN with kernel size 10. Each layer is followed by batch normalization, two ReLU activations, and a dropout layer (rate = 0.01). The model was trained using weighted binary cross-entropy loss, the Adam optimizer (learning rate = 0.001), and early stopping based on validation AUC. Training was conducted with a batch size of 20 for up to 150 epochs. Model performance was assessed using 5-fold stratified cross-validation. We report AUC-ROC, AUC-PR, accuracy, sensitivity, specificity, F1-score, Brier score, and calibration curves to evaluate both discrimination and calibration. All preprocessing, model implementation, and validation were implemented in Python 3.12.3. The filters used in the prepossessing step were obtained from the SciPy 1.12 [27] and NumPy 1.26.4 [10] libraries. The prediction model was implemented using TensorFlow 2.17.0 [1].

## Results

**Fig. 4:**
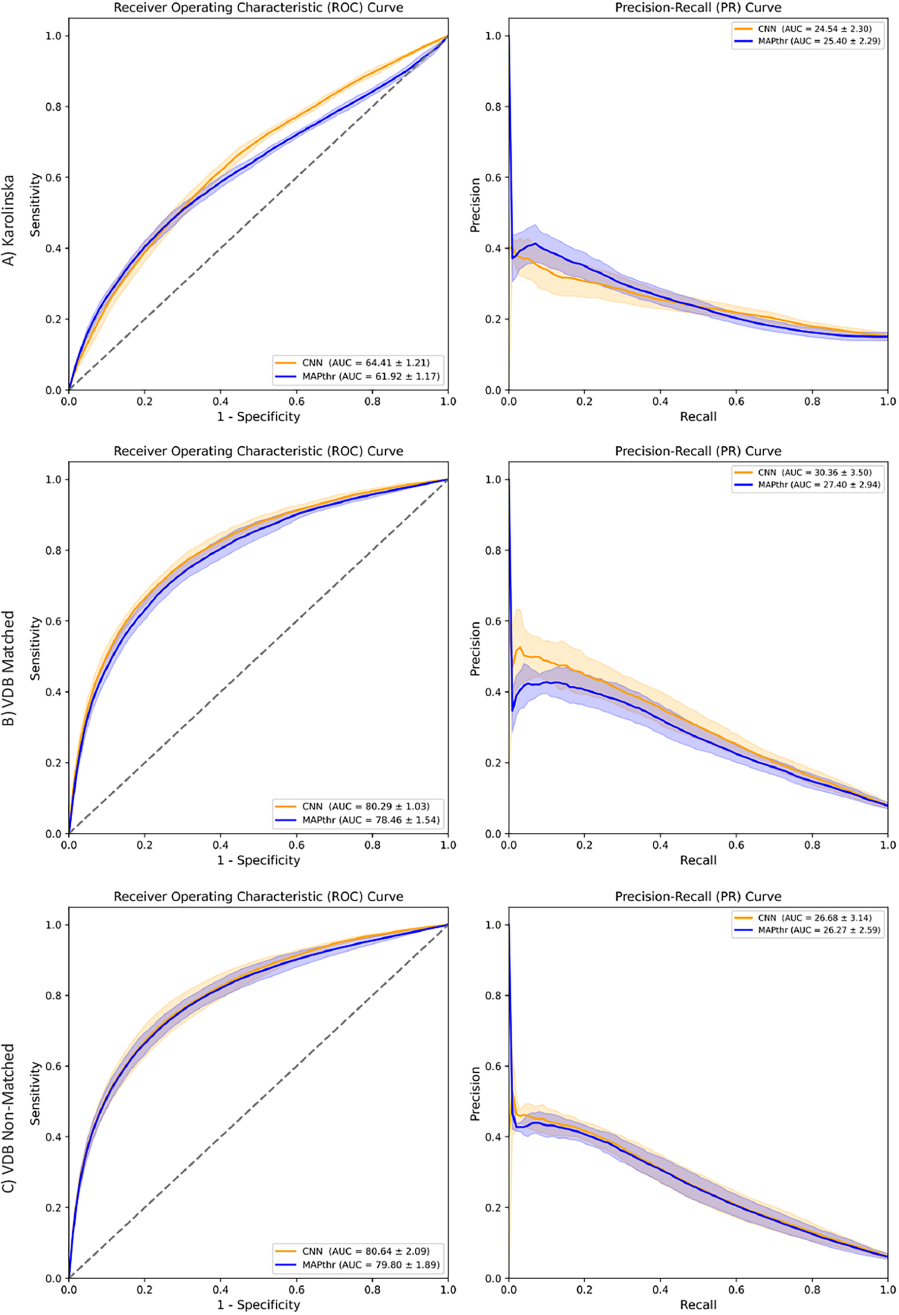
Receiver Operating Characteristic curves (left panel) and Precision-Recall curves (right panel) were constructed for the CNN-based model and MAP threshold method (MAPthr) using A) Karolinska, B) VBD Matched, and C) VDB Non-Matched datasets. In each case, both methods were trained (or applied) and evaluated on the same dataset to assess within-dataset performance for predicting hypotension before the event. Values are shown as mean ± CI (in $\%$)

**Evaluation between CNN-based model and MAP threshold method**: In the Karolinska dataset, characterized by narrow class separation ($\Delta \text {Mean}$ = 2.44 mmHg), the CNN model performed better than MAP threshold method (MAPthr), achieving a higher $AUC_{ROC}$ (64.41$\%$ vs. 61.92$\%$). This demonstrates the CNN model’s advantage in diagnostically ambiguous cases. In contrast, for the VDB datasets–characterized by a wider class separation ($\Delta \text {Mean}$ = 11.21–11.96 mmHg) and clearer hemodynamic patterns–the CNN model performed only marginally better than MAPthr ($AUC_{ROC}$ = 80.29$\%$ vs. 78.46$\%$ for VDB Matched, and 80.64$\%$ vs. 79.80$\%$ for VDB Non-Matched). These results highlight the strength of MAP-based features in less ambiguous scenarios (Fig. 4).

Additionally, the accuracy, sensitivity, F1-score, PPV, and NPV remained comparable between methods across all datasets with a specificity target of approximately 80$\%$ (false positive rate of 20$\%$) (Table 2). This similarity in performance suggests that the CNN model may be predominantly learning from MAP, the major feature in the signal, which effectively aligns its performance with the MAPthr approach. Consequently, our findings underscore the challenge of distinguishing transient drops from sustained hypotension, even with improved data selection methodologies that reduce but do not eliminate inherent biases in hypotension prediction.

**Table 2:** Evaluation metrics for the CNN-based model and MAP threshold method (MAPthr) in predicting hypotension before onset using Karolinska, VDB Matched, and VDB Non-Matched datasets

	CNN	MAPthr
Accuracy		
Karolinska	73.82 ± 0.61	74.04 ± 0.50
VDB Matched	78.93 ± 0.20	78.67 ± 0.29
VDB Non-Matched	79.22 ± 0.26	79.18 ± 0.20
Sensitivity		
Karolinska	38.94 ± 2.56	40.42 ± 1.71
VDB Matched	66.38 ± 1.88	63.06 ± 2.71
VDB Non-Matched	67.06 ± 4.38	66.40 ± 3.08
Specificity		
Karolinska	80.00 ± 0.00	80.00 ± 0.00
VDB Matched	80.00 ± 0.00	80.00 ± 0.00
VDB Non-Matched	80.00 ± 0.00	80.00 ± 0.00
F1 score		
Karolinska	30.87 ± 2.15	31.87 ± 1.80
VDB Matched	32.92 ± 2.40	31.52 ± 2.27
VDB Non-Matched	28.05 ± 3.14	27.81 ± 2.89
False positive rate		
Karolinska	20.00 ± 0.00	20.00 ± 0.00
VDB Matched	20.00 ± 0.00	20.00 ± 0.00
VDB Non-Matched	20.00 ± 0.00	20.00 ± 0.00
Positive predictive value		
Karolinska	25.62 ± 2.15	26.35 ± 2.02
VDB Matched	21.94 ± 2.10	21.06 ± 1.98
VDB Non-Matched	17.78 ± 2.35	17.64 ± 2.23
Negative predictive value		
Karolinska	88.08 ± 1.05	88.34 ± 0.96
VDB Matched	96.55 ± 0.47	96.22 ± 0.58
VDB Non-Matched	97.41 ± 0.46	97.36 ± 0.41

In each case, both methods were applied and evaluated on the same dataset. Values are shown as mean ± CI (in %). Classification cutoff thresholds were selected to achieve an 80% specificity with a 20% false positive rate

**Cross-center and cross-cohort validations**: As outlined in our framework for evaluating generalizability, we tested the model performance across different clinical settings and patient populations. Cross-center validation exposed asymmetric generalizability: models trained on Karolinska–with a larger Gray Zone prevalence ($\approx$ 49 $\%$ versus 18–20 $\%$ in VDB datasets; see Fig. 3, right panels)–generalized moderately to VDB Matched ($AUC_{ROC}$ = 68.28$\%$), while the reverse (VDB Matched $\,\rightarrow \,$Karolinska) showed significant degradation ($AUC_{ROC}$ = 63.25$\%$). Similarly, cross-cohort validation revealed variability: Karolinska$\,\rightarrow \,$VDB Non-Matched achieved $AUC_{ROC}$ = 70.62$\%$, whereas VDB Non-Matched$\,\rightarrow \,$Karolinska fell to 63.76$\%$. However, models trained and tested within the VDB cohort (Matched$\leftrightarrow$Non-Matched) achieved consistent performance ($AUC_{ROC}$ = 79.16–80.63$\%$), demonstrating reliability when surgical procedures and hemodynamic profiles align. This asymmetry directly addresses our concern regarding limited external validation in previous studies and highlights that generalizability depends significantly on dataset similarity–particularly the extent of borderline (Gray Zone) cases–a critical insight for translating hypotension prediction models into diverse clinical environments.

**Table 3:** The average test $AUC_{ROC}$ values (mean ± 95% CI in $\%$) of the CNN-based model among all datasets

Train	**Test**		
	Karolinska	VDB Matched	VDB Non-Matched
Karolinska	64.41 ± 1.21	68.28 ± 4.67	70.62 ± 4.41
VDB Matched	63.25 ± 1.40	80.29 ± 1.03	80.63 ± 1.78
VDB Non-Matched	63.76 ± 1.60	79.16 ± 1.39	80.64 ± 2.09

**Table 4:** The average test $AUC_{ROC}$ values (mean ± $95\%$ CI in $\%$) of the CNN-based model between age cohorts

**Train**	**Test**	
	Old	Young
Old	74.86 ± 1.02	80.19 ± 1.65
Young	75.01 ± 1.29	80.94 ± 1.69

**Cross-age, mean MAP, and ASA classification testing**: Addressing the demographic variability concerns identified in our framework, we evaluated model performance across age groups, MAP distributions, and ASA classifications. For cross-age testing, the CNN-based model consistently achieved higher AUC values when tested on the Young cohort ($AUC_{ROC} = 80.19\%$ for Old-trained and $AUC_{ROC} = 80.94\%$ for Young-trained) compared to testing on the Old cohort ($AUC_{ROC} = 74.86\% \; \mathrm{and} \; AUC_{ROC} = 75.06\%$, respectively), as shown in Table 4. This suggest that age-related factors may introduce variability or complexity in the data for older patients, potentially making accurate prediction more challenging. 

 To address the methodological bias concerns, we standardized the mean MAP difference ($\Delta \text {Mean}$) to 5.01 mmHg across all datasets. Fig. S3 in the Supplementary Materials (Section 5) shows the resulting mean MAP distributions for positive and negative samples (measured before hypotension/non-hypotension events), illustrating the effect of this standardization. This standardization equalized performance ($AUC_{ROC} \in [71.79-73.33]$ for Karolinska, VDB Matched, and VDB Non-Matched), confirming that class separation bias inflates metrics in datasets with larger $\Delta \text {Mean}$ values (Table 5). This finding directly validates our approach of redefining non-hypotension events to include borderline MAP values and demonstrates how selection bias impacts model evaluation.

Finally, when restricting analysis to ASA class 2 only patients, the model maintained comparable performance to the baseline across all datasets ($\Delta AUC_{ROC} < 0.01$). This robustness across patient fitness levels suggests that ASA classification, while clinically significant, does not substantially impact model reliability in our framework.

**Table 5:** CNN performance of controlled mean MAP distributions ($\Delta \text {Mean}$ = 5.01 mmHg) and ASA classification = 2 in comparison with baseline. SD = Standard deviation

Patients characteristic	Dataset	$AUC_{ROC}$ (Mean ± CI in $\%$)
Baseline	Karolinska	64.41 ± 1.21
	VDB Matched	80.29 ± 1.03
	VDB Non-Matched	80.64 ± 2.09
Standardized mean MAP distributions	Karolinska	72.48 ± 1.63
	VDB Matched	71.79 ± 0.98
	VDB Non-Matched	73.33 ± 1.33
ASA classification = 2	Karolinska	62.93 ± 2.45
	VDB Matched	79.33 ± 2.68
	VDB Non-Matched	79.46 ± 1.53

## Discussion

Our findings reveal a central tension in AI-driven hypotension prediction: complex models like CNNs add value in uncertain, real-world scenarios but offer no advantage over simple thresholds in straightforward cases. By including the Gray Zone–often excluded in prior studies–we reduced inflated performance metrics and showed how dataset design shapes AI reliability. For example, models trained on Karolinska data (rich in borderline cases) adapted better to VDB datasets than vice versa, underscoring the importance of diverse training data.

### Comparison between methods

Our analysis reveals that the CNN model performed better than the MAP threshold approach in ambiguous cases–such as in the Karolinska dataset with narrow class separation and considerable hemodynamic ambiguity–where subtle waveform nuances appear critical. In the VDB datasets with larger MAP differences, both methods achieved comparable results. This convergence in performance suggests that deep learning models primarily extract MAP-related features in clear-cut scenarios, aligning their predictions with straightforward MAP thresholds when hypotension precursors are evident. However, the CNN’s advantage in diagnostically uncertain cases–where its ability to capture nuanced hemodynamic patterns proves most valuable–must be interpreted cautiously.

Our inclusion of transient MAP drops (<65 mmHg for <1 minute) and the Gray Zone reduced selection bias compared to prior studies that applied stricter or arbitrary thresholds for defining negative cases. By avoiding a lead window and implementing a Poisson process-based sampling strategy with a slack window, our design prevents artificial separation between positive and negative classes, which often leads to over-optimistic performance. This approach mitigates data leakage by ensuring that the observation window is entirely hypotension-free and temporally decoupled from the event onset. Furthermore, the design is forward-looking–thus avoiding backward anchoring on known events and preserving the natural variability of the hemodynamic signal, including borderline MAP values, thereby promoting a more clinically realistic and unbiased evaluation of model performance.

### Cross-center and cross-cohort differences

Our cross-validation results show that generalizability in hypotension prediction work best when tested in settings similar to where they were trained–a key insight for real-world use. The asymmetric performance demonstrates that model reliability depends critically on alignment between training and clinical environments. Two key determinants emerged: exposure to ambiguous blood pressure patterns during training and similarity in class separation trends between source and target populations.

Models trained on datasets with high Gray Zone cases developed robustness that transferred well to more clearly separated cohorts, while those trained on well-separated data struggled with borderline cases. This asymmetry represents a novel insight extending beyond hypotension prediction to broader questions of AI reliability in heterogeneous healthcare environments.

The strong single-center performance but variable cross-center transfer reflects clinical reality–where protocols, populations, and practices vary significantly. These findings establish realistic expectations for AI-deployment in one hospital doesn’t guarantee success in another. By quantifying generalizability boundaries, our framework moves reliability assessment beyond single-center validation, addressing a critical gap in clinical AI evaluation.

### Demographic and physiological influences on model reliability

Our examination of demographic and physiological factors revealed that age appears to influence model performance, with younger patients showing more predictable patterns that the CNN can capture effectively. In contrast, older patients may exhibit more complex or variable physiological signals, which can make accurate prediction more difficult. These observations underscore the value of including data from a broad age range to enhance the generalizability of predictive models. 

The equalization of performance metrics after standardizing mean MAP differences confirms that class separation bias fundamentally influences reported predictive performance, validating our approach of including borderline MAP values. This suggests that many reported variations in model efficacy may reflect dataset characteristics rather than algorithmic superiority.

The reliability of model performance across ASA classifications indicates that patient fitness does not substantially alter the hemodynamic trends predicting impending hypotension. This robustness to comorbidity status is encouraging for clinical implementation, suggesting models may maintain reliability across diverse fitness levels. Together, these analyses provide a multidimensional view of reliability that considers how patient characteristics influence predictive value.

### Study limitations

Our study acknowledges some limitations that pave the way for future research. First, our datasets lack detailed records of intraoperative interventions (e.g., vasopressor administration, fluid resuscitation, or blood loss), which may influence blood pressure trends and limit model interpretability.

Additionally, while we employed a generalized CNN-based classifier to ensure applicability across heterogeneous datasets, this approach may not fully capture the nuances of specific patient subgroups or surgical contexts. Tailored models–optimized for high-risk populations, specific procedures, or clinical environments–may yield improved predictive performance in targeted applications. Investigating such context-adaptive models represents a promising direction for future work.

Moreover, the inherent limitation of classification approaches lies in their dependence on threshold-based labeling, which may oversimplify the dynamic and continuous nature of intraoperative hemodynamics. This dichotomization can obscure subtle physiological trends and contribute to reduced temporal precision in prediction. Exploring time-to-event models or continuous outcome forecasting could provide more actionable and clinically interpretable insights.

Finally, expanding validation efforts to include multimodal datasets–incorporating ECG, fluid balance, and medication records–could enhance generalizability across diverse clinical workflows and improve the robustness of predictive models in real-world deployment.

## Conclusion

Predicting intraoperative hypotension requires balancing technical innovation with reliable clinical validation. Our findings suggest that the CNN model demonstrates improved performance in ambiguous hemodynamic states but performs similarly to simpler methods in clear cases. By including borderline blood pressure values and testing across hospitals and surgical cohorts, we exposed biases in earlier studies and demonstrated that diversity in training data is non-negotiable for real-world use. While generalizability is essential, tailoring models to specific patient populations may offer additional performance gains in targeted settings. Future work should focus on refining prediction strategies by forecasting continuous MAP trajectories, reducing prediction windows to capture acute hypotensive events more precisely, integrating intraoperative intervention data (e.g., vasopressor administration), and investigating whether condition-specific or context-adaptive models can enhance performance in high-risk subgroups. Ultimately, the successful translation of AI-driven hypotension prediction into clinical practice will depend on aligning algorithmic sophistication with the complex realities of perioperative hemodynamic management.

## Supplementary Information

Below is the link to the electronic supplementary material.

Supplementary file 1 (pdf 4909 KB)

## Data Availability

Ethical permits for sharing or distribution of the Karolinska data was neither requested nor granted, due to its sensitive nature. The Karolinska dataset is therefore not available upon request from the authors. The VitalDB dataset is openly available through their website.
