# Supplementary material for: Towards reliable prediction of intraoperative hypotension: a cross-center evaluation of deep learning-based and MAP-derived methods
Source: J Clin Monit Comput. 2025 Sep 12;40(1):43–57. doi: 10.1007/s10877-025-01357-0 (PMC12963106; doi:10.1007/s10877-025-01357-0)
Supplement: Supplementary file 1 — (pdf 4909 KB) [file 10877_2025_1357_MOESM1_ESM.pdf]

Supplemental Information: Towards Reliable Prediction of Intraoperative Hypotension - A Cross-Center Evaluation of Deep Learning-Based and MAP-Derived Methods

S1 Data preprocessing

Table S1 Used limit criteria for valid ABP pulses.

Feature	Limit
ABP	$30 \leq \text{ABP} \leq 250$ mmHg
MAP	$35 \leq \text{MAP} \leq 150$ mmHg
PP = SBP − DBP	PP ≥ 15 mmHg
T = beat duration	$0.2 \leq T \leq 6$ seconds

The data preprocessing steps aim to clean and prepare the patient's ABP waveforms for subsequent data selection. A rigorous preprocessing pipeline was implemented, including the following key steps: 1) Noise removal: We remove data segments with extreme values outside the physiologically plausible range for ABP (≤ 30 mmHg or ≥ 250 mmHg). 2) Peak detection and artifact cleaning: We identify systolic (SBP) and diastolic blood pressures (DBP) within the ABP waveform using an automatic peak detection method based on Gaussian derivative filters and the Shannon energy envelope [2]. Subsequently, a beat-to-beat noise and artifact detection algorithm removes outliers based on a signal abnormality index. This index compares waveform features like SBP, DBP, pulse pressure, and beat duration against established physiological limits and inter-beat variability. Additionally, methods to detect high-frequency noise and square waves are employed [3, 4]. Table S1 contains the physiological limits used to include valid beats. 3) Mean arterial Pressure (MAP) calculation: Using the identified DBP peaks, we mark the onset of each beat for MAP calculation. MAP is then derived by averaging the ABP values within each beat. Beats with non-physiological MAP values (outside 35-150 mmHg) are excluded. 4) Data filtering: Finally, for each patient, we filter based on the percentage of clean ABP data available. Patients with less than 90% clean ABP data across the entire surgery duration are excluded from all datasets (Karolinska, VitalDB Matched, and VitalDB

Non-Matched). The ABP signals from the VitalDB dataset was preprocessed in the same way. However, since the sampling frequency of the ABP is 500 Hz, we first down-sampled the signal to 125 Hz using a discrete Fourier transform-based resampling method before applying the preprocessing.

S2 Data point selection

The correlation between the mean MAP values of hypotension and non-hypotension events and their corresponding positive and negative data points, extracted 5 minutes prior to each event, across the Karolinska, VDB Matched, and VDB Non-Matched datasets. The x-axis represents the mean MAP of the data points, while the y-axis shows the mean MAP at the time of the event. The mean MAP for both data points and events is calculated by averaging the MAP values over the respective time windows: 20 seconds for data points and the first minute of the event. The latter explains why some non-hypotension event mean MAP values fall below the 65 mmHg threshold, as the 1-minute window may intersect with a brief hypotensive period lasting less than one minute. We include non-hypotension events with mean MAP values, particularly within the 65-75 mmHg range, for each dataset, see Fig. S1.

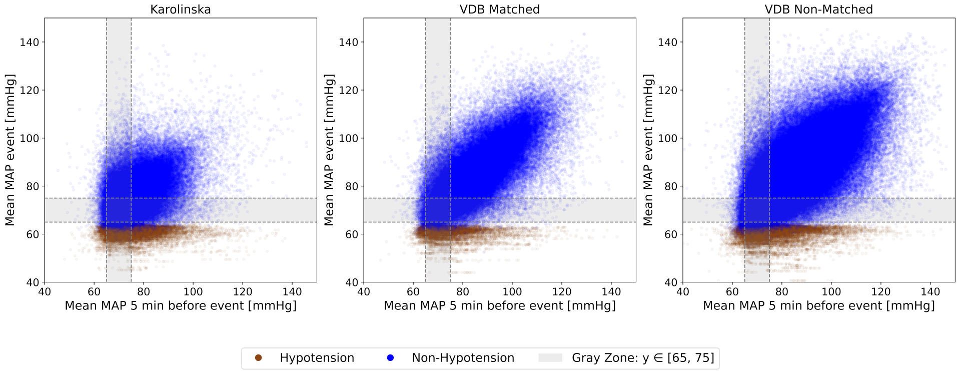

Fig. S1 Visualization of data selection for classifier training and validation. This includes hypotension and non-hypotension events, along with their corresponding positive and negative data points, extracted 5 minutes prior to the event onset. The y-axis represents the mean MAP of the hypotension and non-hypotension events, while the x-axis shows the mean MAP of the positive and negative data points, which are used for prediction 5 minutes before the event. Unlike most prior work, non-hypotension events include the Gray Zone (65–75 mmHg) and transient MAP <65 mmHg (lasting <1 minute), eliminating artificial separation between classes. Hypotension events (brown) are strictly MAP <65 mmHg (sustained $\geq$ 1 minute). Non-hypotension events (blue) include MAP values $\geq$ 65 mmHg and transient dips below 65 mmHg, reflecting clinically realistic ambiguity. This approach reduces selection bias inherent in earlier studies (e.g., [1]).

S3 Model architecture and training

The CNN-based model consists of seven convolutional layers with a kernel size of 10. Each CNN layer is followed by a batch normalization, two layers of rectified linear

Table S2 Model and training parameters

	Parameters	Value
Data	Pred_t (minutes)	5
	Δt (seconds)	20
	λ (minutes^{-1})	3
Classifier	Batch size	256
	Learning rate	$5 \cdot 10^{-4}$
	Dropout	0.6
	No. of epochs	150
	No. CNN Layers	7
	Loss function	Weighted binary cross-entropy
	Optimizer	Adam optimizer

activation unit for non-linearity, and a Dropout layer (rate of 0.01) was connected to prevent overfitting of the classification task. We train the classifier with a batch size of 256 and we use the weighted binary cross-entropy as a loss function and the

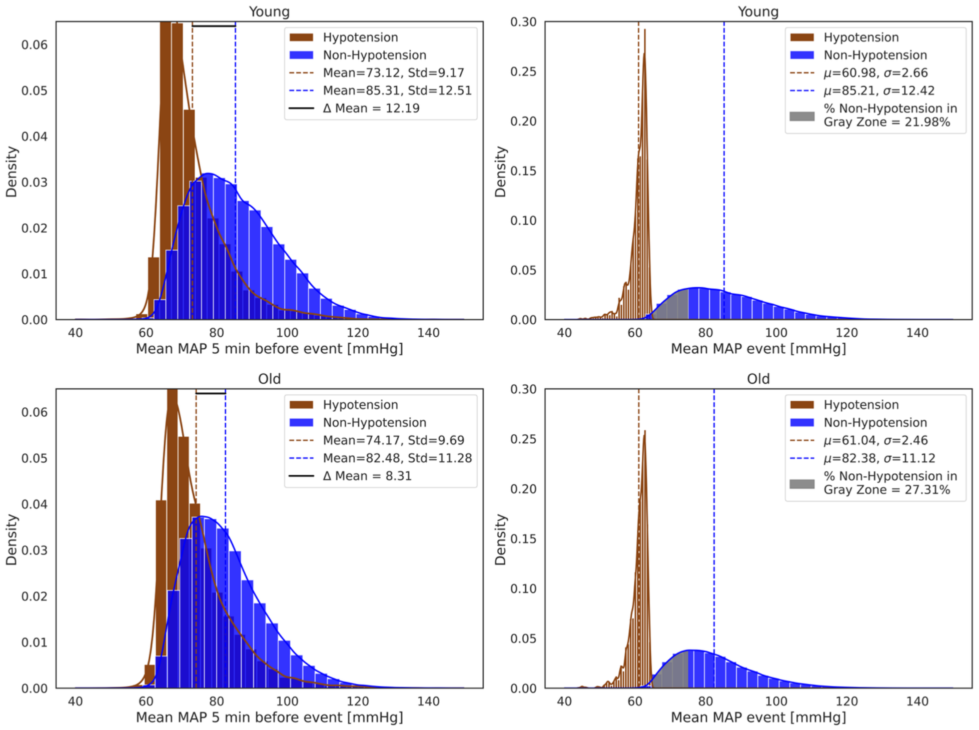

Fig. S2 On the left side of the figure is the distribution of the mean MAP values for positive and negative 5 minutes before the onset of hypotension and non-hypotension events. On the right side is the distribution of the mean MAP values during hypotension and non-hypotension events for the Old and Young cohorts.

Table S3 Patient characteristics for the young and old cohorts. Values shown as mean ± standard deviation), or as count (fraction in %)

dataset	Young cohort	Old cohort
Number of patients	756	782
Age (years):	44.09 ± 9.41	74.3 ± 4.62
Sex		
Male	382 (50.5%)	491 (62.8%)
Female	374 (49.5%)	291 (37.2%)
Height (cm)	165.94 ± 8.85	163.06 ± 9.48
Weight (kg)	64.5 ± 13.9	63.26 ± 13.28
BMI	23.29 ± 3.99	23.66 ± 3.72
ASA class		
1.0	254 (33.6%)	51 (6.5%)
2.0	372 (49.2%)	529 (67.5%)
3.0	112 (14.8%)	185 (23.7%)
4.0	3 (0.4%)	10 (1.3%)
Hypertension	136 (18.0%)	420 (53.7%)
Diabetes	43 (5.7%)	151 (19.3%)
Pulmonary disease	93 (12.3%)	219 (28.0%)
Hemoglobin (g/l)	128.71 ± 21.89	125.56 ± 17.69
Glucose (mmol/l)	5.99 ± 1.72	7.13 ± 2.95
Albumin (g/l)	40.61 ± 5.37	38.15 ± 5.02
Creatinine (micromol/l)	142.28 ± 252.66	84.07 ± 54.13
Sodium (mmol/l)	139.82 ± 2.94	139.88 ± 2.87
Potassium (mmol/l)	4.15 ± 0.43	4.17 ± 0.38
Department		
General surgery	558 (73.7%)	532 (68.0%)
Thoracic	108 (14.3%)	185 (23.7%)
Gynecology	76 (10.1%)	33 (4.2%)
Urology	14 (1.9%)	32 (4.1%)
Approach		
Open	389 (51.5%)	424 (54.2%)
Videoscopic	306 (40.5%)	323 (41.3%)
Robotic	61 (8.0%)	35 (4.5%)
Elective surgery	670 (88.6%)	736 (94.1%)
Total surgery duration (h):	3.9 ± 1.61	3.98 ± 1.83
Mean MAP (mmHg)	82.48 ± 10.47	80.39 ± 8.52
Hypotension prevalence	403 (53.3%)	544 (69.6%)
Number of hypotension events	4.03 ± 6.95	4.69 ± 6.64
Total hypotension duration (min)	14.85 ± 29.99	15.76 ± 26.75
Time fraction in hypotension (%)	6.32 ± 12.13	6.59 ± 10.0
Hypotension event duration (min)	1.74 ± 2.1	2.12 ± 1.91
AUT (mmHg·min)	9.64 ± 13.68	11.64 ± 12.82
TWA (mmHg)	0.05 ± 0.07	0.06 ± 0.07
ΔMean (mmHg)	18.74	13.38

BMI = Body mass index, ASA = American Society of Anesthesiologists, MAP = Mean arterial pressure, AUT = Area under threshold, TWA = Time-weighted average, ΔMean = The difference between the mean MAP values of the positive and negative data point distributions.

Adam optimizer to minimize the loss with an initial learning rate of 0.01). The training process was repeated until either 150 epochs of iterations had been completed

or convergence had been achieved. The final model was then selected based on the best validation results. We feed our time series datasets corresponding to the preprocessed ABP signals into the CNN-classifier which learns in a supervised manner the classification of positive and negative data points representing hypotensive and non-hypotensive events given the class labels $y = 1$ and $y = 0$, respectively. We set the classifier hyperparameters using a random grid search method to find the combination given the best performance results. We trained the classifier for a maximum of 150 epochs, with early stopping applied based on validation performance to prevent overfitting. The sets of hyperparameters used for the CNN classifier are presented in Table S2.

S4 Old Young Demographics

Figure S2 shows the distribution of mean MAP values for positive and negative data points across the old and young datasets. Table S3 summarizes key demographics and clinical characteristics for the old and young datasets.

S5 Standardized Mean MAP distributions

To ensure consistent class separation across all datasets and allow fair comparisons of model performance, we applied a stratified sampling approach based on MAP values: the full MAP range was divided into 100 equal-width bins (1 mmHg each), and for each bin and class (positive/negative), we identified the minimum number of samples available across the three datasets. Each dataset was then randomly sampled to match this minimum per class and bin.

This bin-wise, class-balanced sampling yielded a standardized ΔMean of approximately 5.01 mmHg across datasets, see Fig. S3. Prior to this process, ΔMean varied widely—from 2 mmHg in Karolinska to 11.96 mmHg in VBD Non-Matched—making within-dataset results difficult to compare. Standardizing ΔMean ensured that performance metrics reflected model behavior under equivalent class difficulty.

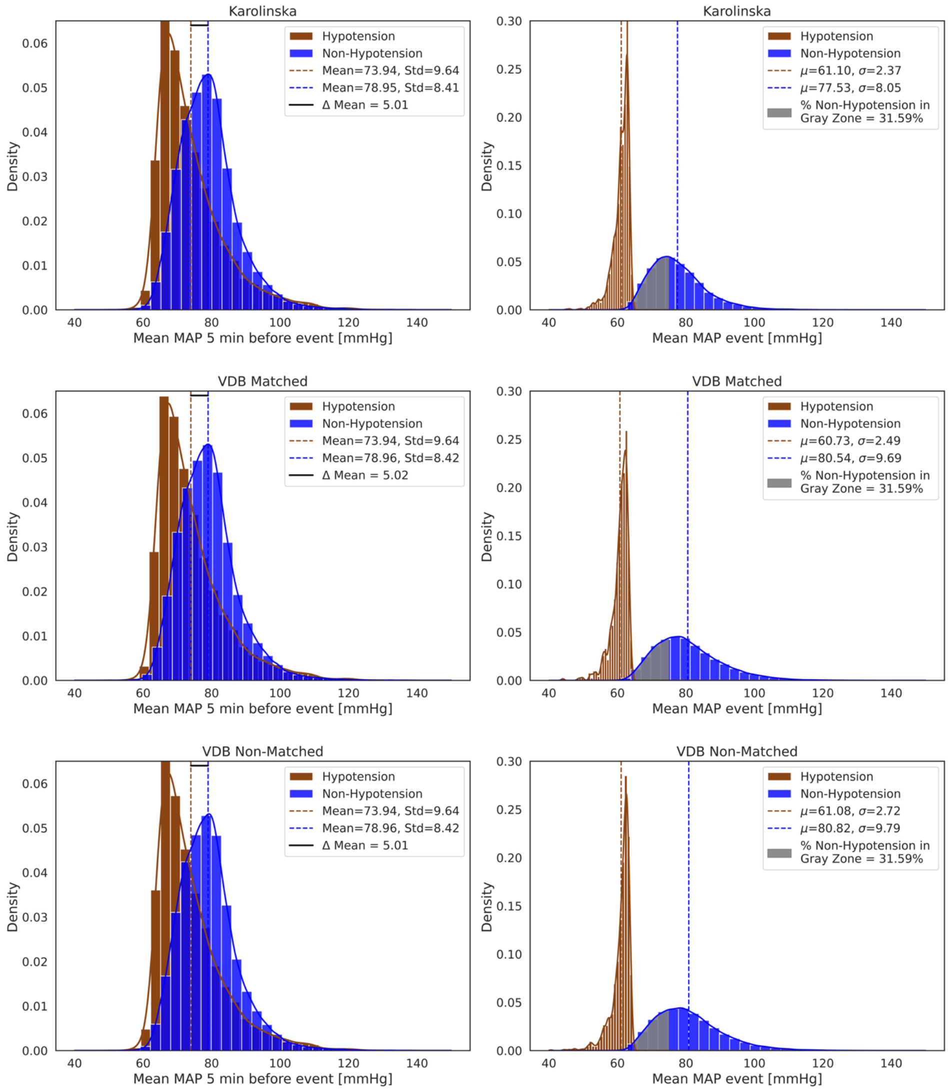

Fig. S3 Left panels: Distribution of mean MAP values for positive and negative data points extracted at the prediction time for hypotension and non-hypotension events, across the Karolinska, VDB Matched, and VDB Non-Matched datasets after the standardization process. Right panels: Distribution of mean MAP values during hypotension and non-hypotension events for the same datasets, also after standardization.

References

[1] Hatib F, Jian Z, Buddi S, et al (2018) Machine-learning algorithm to predict hypotension based on high-fidelity arterial pressure waveform analysis. Anesthesiology (Philadelphia) 129(4):663–674

[2] Raju DS, Manikandan MS, Barathram R (2014) An automated method for detecting systolic peaks from arterial blood pressure signals. In: Proceedings of the 2014 IEEE Students' Technology Symposium, IEEE, pp 41–46

[3] Sun J, Reisner A, Mark R (2006) A signal abnormality index for arterial blood pressure waveforms. In: 2006 Computers in Cardiology, IEEE, pp 13–16

[4] Zhang P, Liu J, Wu X, et al (2010) A novel feature extraction method for signal quality assessment of arterial blood pressure for monitoring cerebral autoregulation. In: 2010 4th International Conference on Bioinformatics and Biomedical Engineering, IEEE, pp 1–4
